# Decoding Surgical Complexity: Measuring the Impact of Operative Difficulty on Quality Outcomes Following Hepatectomy for Liver Cancer over Two Decades

**DOI:** 10.3390/cancers18030407

**Published:** 2026-01-27

**Authors:** Meet Patel, Jonathan Ben Daniel, Nazim Bhimani, Anthony R. Glover, Thomas J. Hugh

**Affiliations:** 1 Faculty of Medicine and Health, The University of Sydney, Science Rd, Sydney, NSW 2050, Australianazim.bhimani@sydney.edu.au (N.B.); anthony.glover@sydney.edu.au (A.R.G.); tom.hugh@sydney.edu.au (T.J.H.); 2 Concord Repatriation General Hospital, Sydney, NSW 2139, Australia; 3 Northern Clinical School, The University of Sydney, Sydney, NSW 2050, Australia; 4 Upper Gastrointestinal Surgical Unit, Royal North Shore Hospital, Sydney, NSW 2065, Australia

**Keywords:** liver, surgery, outcomes, oncology, risk prediction, difficulty

## Abstract

**Introduction**: Operative time is commonly used as a surrogate marker for operative difficulty in liver resection, but the contribution of other intraoperative factors is less understood. This study aimed to develop an objective, composite score to assess operative difficulty and evaluate its association with postoperative and oncological outcomes in liver surgery. **Methods:** A retrospective cohort study was conducted on patients who underwent liver resection for malignant disease between 1999 and 2023 at an Australian tertiary hospital, using a prospectively maintained database. Principal component analysis (PCA) was applied to operative time, estimated blood loss, total time of hepatic inflow occlusion and the number of packed red bloods transfused intraoperatively to derive a composite operative difficulty score. Patients were then stratified into low, moderate and high difficult groups using Gaussian mixture models (GMM). Comparison of textbook oncological outcomes (TOO) achievement and futile resection rates were assessed using Chi-squared analysis. Kaplan-Meier analysis was used to assess recurrence-free and overall survival in subgroup analysis. **Results:** Of 729 patients, 699 met the inclusion criteria. GMM identified three distinct operative difficulty groups: low (n = 540), moderate (n = 143), and high (n = 16). TOO and non-futile resection rates declined with increasing difficulty: 77% and 58% (low), 47% and 52% (moderate), and 6% and 19% (high), respectively (*p* < 0.001, *p* = 0.004 respectively). Among patients with cholangiocarcinoma, median overall survival was inversely correlated with operative difficulty (40 months low, 16 months moderate, 7 months high, *p* = 0.004). In patients with colorectal liver metastases, there was a trend towards worse overall survival and disease-free survival with increasing operative difficulty, however, this did not reach statistical significance. **Conclusions:** An objective intraoperative difficulty score was developed and demonstrated a significant inverse association with both quality and oncological outcomes. While external validation is required, these findings support the potential of operative difficulty assessment to enhance perioperative decision-making, inform patient counselling, and optimise postoperative care planning.

## Simple Summary

Liver surgery is complex and currently there are significant inconsistencies in determining the difficulty of a procedure. Most existing tools rely on operative time alone, which may not reflect the experience of the operator or unexpected challenges during surgery. This retrospective study aimed to develop an objective operative difficulty score using intraoperative variables (operative time, estimated blood loss, time of hepatic inflow occlusion, and the need for blood transfusion) and stratify patients into low, moderate and high operative difficulty groups. A prospectively collected liver resection database was used, and 699 patients were included in the study. As the operative difficulty score increased, surgical quality and cancer outcomes worsened. Patients in the high operative difficulty group had much lower rates of textbook oncological outcomes and higher rates of futile surgery. Among patients with cholangiocarcinoma and colorectal liver metastases, greater difficulty was also linked to shorter survival and earlier cancer recurrence. Measuring operative difficulty in this way may improve patient counselling, postoperative planning, and quality assessment after liver surgery.

## 1. Introduction

Resection is indicated in selected patients with primary or secondary liver cancers, where complete excision with clear surgical margins provides the best chance for long-term survival. Primary liver tumours are the sixth most common cause of cancer, with the dominant histological subtype being hepatocellular carcinoma (HCC), followed by cholangiocarcinoma [1]. There are ≥750,000 deaths per year from primary liver cancer worldwide [1]. The liver is also a common site of metastatic disease, particularly from primary colorectal tumours, and select patients can benefit from resection of these metastases [2].

The spectrum of operative difficulty in liver surgery is broad and depends on the extent of the resection as well as any underlying local or systemic disease. Hepatic surgery varies from simple wedge resections to extended or posterior non-anatomical hepatectomies, and procedures requiring biliary or vascular reconstructions increase the technical difficulty [3]. To date, assessment of the impact of operative difficulty have focussed on operative time as a surrogate marker for operative difficulty in hepatic surgery. For example, Lee et al. (2016) classified procedures defined by the Brisbane nomenclature system into three operative difficulty groups for open liver resection based on expert opinion while the Institut Mutualiste Montsouris (IMM) score for laparoscopic liver resection proposed by Kawaguchi et al. (2018) was developed using outcomes based on a retrospective review of a prospectively maintained database [4,5]. Both were validated using operative time and estimated blood loss (EBL) as surrogate markers of difficulty [4,5,6]. Other predictive scoring tools for operative difficulty such as the DIFF-sCOR, the IWATE difficulty score, the Southampton difficulty scoring system, or the Hasegawa scoring system were also developed or validated using operative time as a surrogate marker of operative difficulty [7,8,9,10,11]. Unfortunately, operative time in isolation may not wholly reflect the true technical challenges of a procedure, with likely differences between high-volume centres with experienced surgeons and teaching hospitals with a range of operators at different stages of the surgical learning curve. Instead, a composite measure, using two or more component outcomes might be beneficial as this might provide a more complete assessment of operative difficulty [12]. Composite measures are increasingly used across healthcare as they improve interpretability, increase statistical efficiency, and enable researchers to determine a net clinical benefit without having to choose a single outcome [12].

Preoperative anticipation of operative difficulty has been shown to correlate with technical difficulty, but there has not been a similar correlation with postoperative morbidity or mortality [13]. In addition, previously described predictive operative difficulty tools, although helpful in predicting intra-operative outcomes, are generally more limited in predicting postoperative outcomes. In laparoscopic difficulty scoring systems, several validation studies have shown that major postoperative complications correlate with increasing predicted operative difficulty [14,15,16]. However, others have shown that these may not be as useful for discriminating minor morbidity when comparing straightforward with more difficult groups [17,18]. Similarly, when considering quality liver outcomes, predictive operative difficulty scoring tools are good at delineating between low and high risk operations, but these are not as helpful for moderate risk groups [18]. In laparoscopic surgery to date, predictive difficulty tools do not effectively discriminate between moderate and high risk in terms of overall and disease-free survival [15]. These tools often consider patient, disease and planned procedure factors to predict the operative difficulty, but, collectively these have not correlated with either short or long-term outcomes. An important consideration is the specific operation performed, which can deviate from the planned procedure because of unexpected challenges or unusual anatomy. Assessment of quality outcomes post-liver resection would be improved by correcting for the actual operative difficulty. Furthermore, using long-term outcomes from a prospectively developed liver resection database, it is hypothesised that a composite assessment of intraoperative difficulty is inversely related to both short- and long-term patient outcomes.

The aim of this study is to develop an objective assessment of intraoperative difficulty for liver resection by using intraoperative parameters to create a composite measure and stratify patients into graded operative difficulty groups. Risk groups will be correlated with postoperative outcomes to develop a quantifiable measure of surgical complexity that complements existing risk assessment.

## 2. Methods and Material

### 2.1. Ethics and Consent

Ethics approval was granted by the Northern Sydney Local Health District Human Research Ethics Committee in accordance with the National Statement on Ethical Conduct in Human Research, 2023 (Approval no: 2024/ETH00274). Prospective data collection for the original database was conducted under ethics approval granted by the Northern Sydney Local Health District Human Research Ethics Committee (Approval no: 2019/ETH12206).

### 2.2. Participants

A retrospective review of patients who underwent liver resection at Royal North Shore Hospital and North Shore Private Hospital (Sydney, Australia) by a single, hepato-pancreatico-biliary (HPB) surgeon (TJH) was undertaken. Data were extracted from a prospective database of consecutively completed liver resections. The inclusion criteria were patients who underwent a potentially curative liver resection for malignancy between June 1999 and August 2023 and aged ≥18 years old. Patients who underwent a palliative resection, resection for benign disease, a diagnostic laparoscopy only, liver biopsies only, or procedures that were abandoned due to metastatic disease at the time of the operation were excluded from the study. Patients with missing data were excluded.

The Clavien-Dindo classification score was used to record peri-operative morbidity up to 90 days and then grouped as either minor (Clavien-Dindo ≤ II) or major (Clavien-Dindo ≥ IIIa) [19]. Peri-operative mortality referred to death during the same admission (in-hospital) or within 90 days of the procedure. Liver-related complications were documented using the International Study Group of Liver Surgery definitions [20,21]. Operative data collected included the type of resection grouped as either minor (≤2 contiguous liver segments) or major (≥3 contiguous liver segments) resection.

TOO were defined as the absence of seven criteria described by Gorgec et al. (2023) in their consensus paper [22]. These were intraoperative incidents (grades 2 or 3), 90-day postoperative complications (Clavien–Dindo III or higher), 90-day readmission due to surgery-related complications Clavien–Dindo Grade 3 or higher, postoperative bile leakage (grades B or C), postoperative liver failure (grades B and C), in-hospital and 90-day mortality, and R1 or R2 resection margins [22]. Extended hospital stay was not included as part of the criteria for TOO. Futile resection was defined as all-cause of mortality within 6 months or recurrence of disease within 12 months [23].

### 2.3. Operative Approach

Intraoperative ultrasound was used to confirm the tumour location and relationships with major vascular and biliary structures. Parenchymal transection was performed using the Cavitron Ultrasonic Surgical Aspirator (CUSA) dissector (Integra LifeSciences Corp., Princeton, NJ, USA) under low central venous pressure conditions, and with intermittent inflow occlusion.

### 2.4. Statistical Analysis

Continuous baseline variables that were normally distributed were presented as mean with standard deviation (SD), while non-normally distributed continuous variables were reported as median and interquartile range (IQR). Categorical variables were presented as frequencies with percentages. Principal component analysis (PCA) was completed to derive a composite score of intra-operative difficulty from multiple correlated variables, given there is no singular measure of operative difficulty. PCA aims to reduce the dimensionality across many variables, by finding new variables that are linear functions of those in the original dataset, that successively maximise variance [24]. Therefore, PCA was performed to identify patterns in intraoperative variables that could subsequently be linked to clinical outcomes. The variables selected for PCA were operative time (minutes), estimated blood loss (mL), total time of hepatic inflow occlusion (mins), and number of units packed red blood cells transfused intra-operatively. These surrogate markers of operative difficulty were identified by a literature review described previously [11]. Suitability of the data for PCA was confirmed using Bartlett’s test of sphericity and the Kaiser-Meyer-Olkin (KMO) measure of sampling adequacy. The eigenvalue-one criterion was applied to determine the number of components to retain [25]. The first principal component score was used to develop an operative difficulty score using z-scores (patient value—mean/standard deviation) based on the component weighting. The z-score was used for ease of use in clinical practice and reproducibility. Each patient was then classified using Gaussian mixture models which are a probabilistic model-based clustering technique using Gaussian distribution, enabling the identification of clusters with varying variances and sizes within an overall population [26,27]. Models with one to six components were fitted and compared using the Bayesian Information Criterion (BIC), with the optimal number of components selected as the model with the best (lowest) BIC. A three-component solution was identified as optimal and was therefore used to define low, moderate and high-operative difficulty. Group membership was analysed for associations with clinical outcomes.

Rates of TOO and futile resection were compared with Chi-squared analysis. Logistic regression was performed to determine factors associated with achieving TOO. All univariable models with a *p*-value < 0.20, were included in the multivariable model. Overall survival and recurrence-free survival were calculated using the Kaplan-Meier method and log-rank test was used for comparison between groups. Subgroup analysis was completed for the three most common histological diagnoses.

Statistical analysis was performed using SPSS 30 for Mac (IBM Corp., Armonk, NY, USA) and R 4.5.1 (The R Foundation for Statistical Computing, 2025).

## 3. Results

A total of 729 patients met the inclusion criteria, although 30 patients were excluded due to missing data (n = 5 had missing operative time, n = 25 had missing total hepatic inflow occlusion time). The baseline cohort characteristics of the 699 patients who were included the analysis are detailed in Table 1. The mean age of the cohort was 64.8 years. Most patients underwent open resection (89%) and the underlying pathology was colorectal liver metastases (CRLM) in 60.5% of the cohort. Favourable preoperative baseline factors included 77.8% of patients being ASA 1 or 2, low rates of chronic liver disease (4.7%), chronic obstructive pulmonary disease (COPD) (1.9%), and renal disease (3.4%), reflecting careful patient selection for liver resection. Postoperative outcomes are shown in Table 2. The average operation length was 199 min, and the average hepatic inflow occlusion time was 15.9 min. 69.1% of patients achieved TOO and 55.8% of patients underwent a non-futile resection as per the definition above. The most common reason for not achieving a TOO was an R1 or R2 resection margin followed by a complication grade ≥3a as per the Clavien-Dindo classification. The median postoperative of length of stay in hospital was eight days (IQR five days).

### 3.1. PCA and Development of an Operative Difficulty Grade

A PCA was completed using the time of operation (minutes), the total time of hepatic inflow occlusion (minutes), the total estimated blood loss (mL), and number of units packed red blood cells transfused intraoperatively. KMO measure of sampling adequacy was 0.64, where a score ≥ 0.6 indicates minimum sampling adequacy [28]. The Bartlett’s test of sphericity was ≤0.001 suggesting the null hypothesis of the correlation matrix can be rejected and the data was suitable for PCA. The first principal component explained 52.64% of variance with an Eigenvalue of 2.11. The second principal component had an Eigenvalue of 0.95. Therefore, the first principal component was used to determine an operative difficulty score.

**Figure 1:**
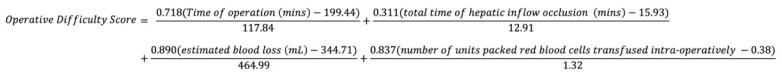
Operative difficulty score equation.

The individual component analysis is seen in Table 3. Using the individual component and z-score, the operative difficulty score was generated using the formula shown in Figure 1. A GMM model was then used to classify the operative difficulty into three latent groups based on this score. The model identified three distinct distributions corresponding to low (n = 540), moderate (n = 143), and high (n = 16) difficulty. Patients were assigned to the group with the highest posterior probability. The cut-off values were:

- Low = score < 0.59
- Moderate = 0.59 ≤ score ≤ 5.15
- High = score > 5.15.

**Table 3:** Component analysis of intra-operative variables.

	Component
Time of operation (minutes)	0.718
Total time of hepatic inflow occlusion (minutes)	0.311
Estimated blood loss (mL)	0.890
Number of units packed red blood cells transfused intra-operatively	0.837

### 3.2. Outcomes Stratified by Operative Difficulty

TOO rate decreased with increasing operative difficulty as seen in Table 4, where patients with low operative difficulty had a TOO rate of 76.9% compared to 46.9% in the moderate difficulty group and 6.3% in the high difficulty group (*p* < 0.001). The rate of futile resection increased with increasing operative difficulty, with a rate of 42% in the low operative difficulty group, compared to 48% in the moderate difficulty group and 81% in the high difficulty group (*p* = 0.004). An increasing Operative Difficulty Score (ODS) was independently associated with worse rates of achieving TOO (odds ratio [OR] 0.66, 95% confidence interval [CI] 0.58–0.75, *p* < 0.001), as seen in the multivariate logistic regression in Table 5. The extent of resection classified by minor/major was the only other variable that was independently associated with achieving TOO, with the results of logistic regression summarised in Table 5. There was no difference in rates of TOO over the 24-year study period.

### 3.3. Long-Term Outcomes Stratified by Operative Difficulty

**Figure 2:**
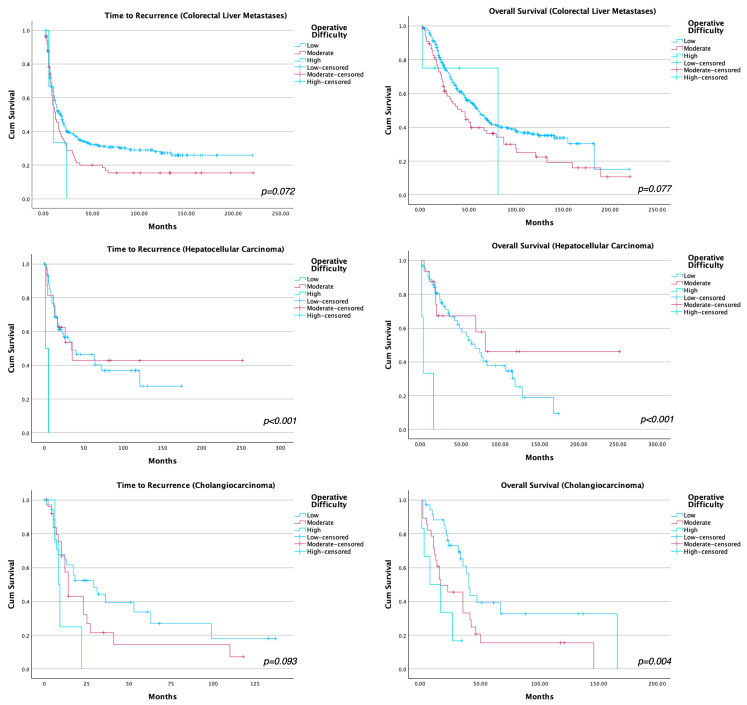
Survival curves for time to recurrence and overall survival stratified by histological diagnosis and operative difficulty.

The median follow-up was 101 months (IQR 29-125) for patients with CRLM, 105 months (IQR 55-138) for patients with HCC and 68 months (IQR 33-133) for patients with cholangiocarcinoma. The results of the survival analysis are summarised in Table 6, and the survival curves are shown in Figure 2. In patients with CRLS, there was a trend towards worse overall-survival (OS) (*p*-value = 0.08) and disease-free survival (DFS) (*p* = 0.07), however, this was not statistically significant. The estimated OS at 14 months was 90.3% in low, 81% in moderate, 50% in high operative difficulty in patients with CRLM.

In the HCC subgroup, no definitive association between operative difficulty and long-term outcomes could be demonstrated, likely reflecting limited statistical power.

In patients with cholangiocarcinoma, operative difficulty was associated with worse OS. There was a difference of 33 months median survival between patients in the low and high operative difficulty groups (*p* = 0.004) as seen in Table 6. There was a trend towards operative difficulty being inversely correlated with DFS in these patients, but this did not reach statistical significance (*p* = 0.09).

## 4. Discussion

In this series of 699 consecutive liver resections, the Operative Difficulty Score enabled objective stratification into low, moderate, and high operative difficulty patients. A clear, graded association was seen between operative difficulty and quality outcomes. Patients in the high-difficulty group had a significantly lower rates of TOO (6% vs. 77% in the low difficulty group), and a higher incidence of futile resection (81% vs. 42% in the low difficulty group). In patients with cholangiocarcinoma, operative difficulty also correlated with worse long-term outcomes, with shorter DFS and OS (29 vs. 8 months and 40 vs. 7 months for high vs. low difficulty, respectively) in more difficult groups. These findings indicate that the proposed ODS captures intraoperative complexity with meaningful links to both perioperative and oncological outcomes. The ODS is easy to calculate and subsequently to classify patients into groups. A simple Excel calculator (Supplementary Material Table S1) facilitates integration into clinical workflows.

A reliable operative difficulty assessment tool plays a valuable role across the perioperative period. Preoperatively, existing tools support surgical planning, consent, and resource allocation by stratifying risk using imaging, comorbidities, tumour location, and surgical history. However, unexpected adhesions, anatomical variations, or bleeding are often only revealed intraoperatively. Structured intraoperative scoring can complement preoperative tools by incorporating real-time operative data, reducing subjectivity, and refining postoperative management. Although, intraoperative scoring occurs after the point of no return for surgical risk, it adds value by enhancing prognostic accuracy, supporting clinical governance, and informing future predictive models. Further validation studies would be required before the ODS can be used as a standardised method of assessing operative difficulty in future studies.

Surgeon assessment of operative difficulty is inherently subjective and often do not to align with postoperative outcomes [13,29]. The treatment of liver cancer requires multidisciplinary input across multiple specialties including, but not limited to, intensive care specialists, medical oncologists, radiation oncologists, gastroenterologists as well as surgeons. As such, it can be difficult to express the nuances of each speciality to one another given varying levels of expertise. Objective measures are needed to guide resource allocation, such as the use of the intensive care unit (ICU). While the role of the ICU after liver resection is debated, it is commonly used for monitoring rather than organ support [30]. An ODS-based approach could identify patients suitable for ward-based care, reducing ICU utilisation and cost [31,32]. Conversely, operations with greater than expected difficulty may warrant enhanced postoperative monitoring or inform the timing of adjuvant therapy. Therefore, utilising an objective ODS as presented in this study is consequential in routine clinical workflows as it can assist with resource allocation and inter-disciplinary communication.

Definitions of futile resection vary, but generally refer to scenarios where procedural risks outweigh benefits [23,33]. In liver surgery, futile resection definitions have encompass recurrence, morbidity, or early mortality [23]. The 44% futile resection rate in this study likely reflects the use of the six-month all-cause mortality as the threshold [23]. Previous studies have linked preoperative tumour factors including number or size, tumour burden index, elevated tumour markers, and disease-specific indicators such as extrahepatic spread, portal vein thrombosis, and neutrophil-to-lymphocyte ratio with futile resection [34,35,36,37,38,39]. Fromer et al. (2022) reported that tumour biology, rather than technical factors, predicted futile hepatectomies, with no difference in margin status between futile and non-futile procedures [39]. While these findings highlight the primacy of tumour biology, our findings could indicate that intraoperative difficulty may serve as a surrogate marker of more extensive disease burden not captured preoperative as seen in the significant difference in futile resection rates between low and high operative difficulty groups. Nonetheless, as shown in the current study by the small (6%) difference in non-futile resections between low and moderate ODS groups, tumour biology likely remains the dominant determinant.

TOO is an “all-or-nothing” composite metric for quality assessment, although exact definitions initially varied [22,40]. Past studies report a median TOO rate of 62% in liver surgery, compared to 54% and 45% in biliary and pancreatic procedures, respectively [41]. The rate of TOO in the present study was 69%, although, markedly lower in the high and moderate ODS group compared to the low ODS group (71% and 30% difference respectively). Although, the high operative difficulty had a low size, the logistic regression showed the ODS was independently associated with decreased chances of achieving TOO, and therefore, this presents an important tool in patient counselling. Since surgeons often hold more optimistic views about outcomes than patients or non-surgeons, aligning expectations is important. An objective ODS can help convey the operative complexity and by communicating the implications of this early, patients can be supported through data-driven informed-decision making and patient preparedness. Importantly, there is evidence that clear prognostic disclosures do not increase distress in patients with advanced cancers so it is important to engage in this type of patient counselling [42]. Therefore, understanding the impact of a difficult operation on the probable postoperative course can aid in expectation management and fostering transparency.

In this study, the ODS was associated with worse OS in patients with cholangiocarcinoma, and there was a trend towards worse DFS. A similar trend was associated in that subgroup analysis of patients with CRLM, but this relationship was not statistically significant in patients HCC, likely due to the small subgroup size and poor statistical power. However, this study suggests a trend towards worse early outcomes with increasing ODS in all liver malignancies. Most prognostic models focus on tumour characteristics and adjuvant treatment, with limited research on the contribution of actual operative complexity [43,44,45,46,47]. Hołówko et al. (2020) reported that a higher preoperative IMM sore in laparoscopic liver resection was associated with increased perioperative complications and significantly worse overall survival, despite similar rates of R0 resection [48]. Given there is limited data on the impact of the actual operative difficulty and long-term survival, the ODS may help standardise future studies aimed at exploring this relationship more robustly.

Limitations of the current study include the retrospective, single-centre design over a 25-year period, during which surgical technique has evolved. For instance, only 11% of resections were laparoscopic, limiting generalisability to contemporary minimally invasive practice. However, this centre is a major teaching hospital, and operations would have taken place from surgeons at all levels of the surgical learning curve which may reduce the impact of this limitation. A further limitation of this study is the small proportion of patients in the high-operative difficulty group (n = 16) which can limit statistical power and precision for long-term outcomes. It is hypothesised that further validation studies can assess whether the current cut-off for operative groups can be adjusted to improve risk prediction, given the relationship determined with logistic regression for TOO and the ODS. Given the exploratory nature of this study, future research should aim for external validation of these findings with retrospective and prospective studies to improve generalisability of the results.

## 5. Conclusions

An objective composite intraoperative difficulty score based on operative time, total time of hepatic inflow occlusion, estimated blood loss, and intra-operative blood transfusion requirement was developed to classify patients into three operative difficulty groups. There was a significant difference among these groups in TOO and non-futile resection rates. In patients with cholangiocarcinoma long-term outcomes oncological outcomes were worse with increasing ODS. Ultimately, integrating operative difficulty tools supports more transparent, data-informed surgical decision-making and helps align expectations between teams and patients. Further external prospective studies are required to validate these findings.

## Figures and Tables

**Table 1:** Baseline cohort characteristics.

	Total n = 699 (%)
Age (mean, SD)	64.8 (11.4)
Gender	
Male	398 (56.9)
Female	301 (43.1)
Diagnosis	
Colorectal liver metastases	423 (60.5)
Hepatocellular carcinoma	83 (11.9)
Cholangiocarcinoma	69 (9.9)
Gallbladder carcinoma	45 (6.4)
Neuroendocrine tumour	23 (3.3)
Other liver metastases	54 (7.7)
Other malignant	2 (0.30)
ASA	
1	277 (39.6)
2	267 (38.2)
3	145 (20.7)
4	10 (1.4)
Cardiac disease	226 (32.3)
Hypertension	137 (19.6)
Ischaemic heart disease	48 (6.9)
Atrial fibrillation	30 (4.3)
Congestive heart failure	1 (0.1)
Previous abdominal surgery	169 (24.2)
Chronic obstructive pulmonary disease (COPD)	13 (1.9)
Renal disease	24 (3.4)
Chronic kidney disease (CKD) stage ≥3	11 (1.6)
Single kidney	5 (0.7)
Other cancers	93 (13.3)
Hepatitis B/C	46 (6.6)
Chronic liver disease	33 (4.7)
Child-Pugh ≥ B	5 (0.7)
Liver cirrhotic intraoperatively	44 (6.3)
Current smoker	111 (15.9)
EtOH ≥100 g/week	171 (24.5)
Previous venous thrombo-emoblism (VTE)	26 (3.7)
Diabetes	107 (15.3)
Non-insulin dependent diabetes	73 (10.4)
Insulin dependent diabetes	34 (4.9)
Elevated tumour markers	418 (59.8)
Neoadjuvant chemotherapy	355 (50.8)
Previous liver resection	75 (10.7)
Open resection	623 (89.1)
Laparoscopic resection	76 (10.9)
Minor resection	425 (60.8)
Major resection	274 (39.2)
Associated biliary anastomosis	46 (6.6)
Associated vascular reconstruction	29 (4.1)

**Table 2:** Postoperative clinical outcomes.

	Total n = 699 (%)
Operation length (mean, SD)	199.4 (117.8)
Whether hepatic inflow occlusion occurred	545 (78.0)
Hepatic inflow occlusion time (mins) (mean, SD)	15.9 (12.9)
Estimated blood loss (mL) (median, IQR)	200 (350)
Whether was transfused intra-operatively	90 (12.9)
Number of units transfused intra-operatively (median, IQR)	0 (0)
Diameter of largest tumour—14 missing (Median, IQR)	38 (43)
Textbook oncological outcomes achieved	483 (69.1)
Intra-operative adverse event ≥grade 2	9 (1.3)
Complication Clavien-Dindo ≥3a	88 (12.6)
Mortality within 90 days or within hospital	20 (2.9)
Bile leak ≥ grade B	26 (3.7)
Postoperative liver failure ≥grade B	26 (3.7)
90-day re-admission for complication Clavien-Dindo ≥3a	5 (0.7)
R1 or R2 resection margin	142 (20.3)
Return to OR	15 (2.1)
Postoperative bleeding	14 (2)
Postoperative bleeding requiring surgical or radiological intervention	2 (0.3)
Cardiac complications	44 (6.3)
Respiratory complications	39 (5.6)
Post operative ileus	19 (2.7)
Wound infections	89 (12.7)
Sepsis	14 (2.0)
Venous thromboembolism	32 (4.6)
Non-futile resection	390 (55.8)
Length of postoperative stay in hospital (days) (median, IQR)	8 (5)

**Table 4:** Comparison operative difficulty group and TOO rates and futile resection rates.

Operative Difficulty Group	Rate of TOO	Rate of Futile Resection
Low (n = 540)	76.9%	42.0%
Moderate (n = 143)	46.9%	48.3%
High (n = 16)	6.3%	81.3%
	*p* < 0.001	*p* = 0.004

**Table 5:** Logistic regression for predictors of achieving TOO.

	Univariate Analysis		Multivariate Analysis	
	OR (95% CI)	*p*	OR (95% CI)	*p*
Operative difficulty score	0.24 (0.17–0.34)	<0.001	0.66 (0.58–0.75)	<0.001
Operation extent		<0.001		0.013
Minor	Reference		Reference	
Major	0.36 (0.26–0.51)		0.60 (0.40–0.90)	
Histopathology		0.025		0.636
HCC	Reference		Reference	
Cholangiocarcinoma	0.50 (0.26–0.96)		0.81 (0.38–1.74)	
Gallbladder cancer	0.87 (0.41–1.88)		0.53 (0.22–1.24)	
Neuroendocrine tumour	1.10 (0.51–3.00)		1.03 (0.30–3.59)	
CRLM	1.26 (0.76–2.09)		1.06 (0.59–1.88)	
Other metastases	1.25 (0.59–2.66)		0.83 (0.36–1.92)	
Year of operation		0.466		0.166
1999–2003	Reference		Reference	
2004–2008	1.30 (0.72–2.36)		1.10 (0.57–2.12)	
2009–2013	1.41 (0.77–2.56)		0.96 (0.49–1.89)	
2014–2018	0.957 (0.525–1.75)		0.60 (0.30–1.17)	
2019–2023	1.25 (0.64–2.42)		0.94 (0.44–2.00)	
Elevate preoperative tumour markers	0.74 (0.53–1.02)	0.071	0.70 (0.48–1.02)	0.064
Previous abdominal surgery	0.73 (0.51–1.06)	0.094	0.78 (0.52–1.18)	0.244
Diabetes	0.74 (0.49–1.15)	0.178	0.83 (0.51–1.37)	0.465
Child-Pugh ≥ B	0.30 (0.04–1.79)	0.183	0.28 (0.04–1.83)	0.184
Cirrhosis	0.89 (0.43–1.94)	0.757		
Previous liver resection	0.72 (0.44–1.20)	0.203		
Hypertension	0.79 (0.53–1.18)	0.243		
Dyspnoea	1.81 (0.57–8.00)	0.362		
Chronic kidney disease	2.03 (0.52–13.40)	0.367		
Neoadjuvant chemotherapy	0.89 (0.65–1.23)	0.481		
Chronic obstructive pulmonary disease	0.71 (0.23–2.38)	0.553		
Ischaemic heart disease	1.22 (0.65–2.44)	0.554		
Age	1.00 (0.99–1.02)	0.803		
Male	Reference	0.870		
Female	0.97 (0.70–1.35)			
Excessive alcohol consumption (≥100 g/week)	1.03 (0.71–1.51)	0.873		
Active smoker	0.97 (0.63–1.51)	0.876		

**Table 6:** Subgroup analysis for recurrence time and overall survival by operative difficulty group.

	Median (95% CI)	*p*-Value
Colorectal liver metastases (n = 423)		
Time to recurrence		
Low (n = 343)	17 (13.1–20.9)	0.072
Moderate (n = 76)	11 (6.1–15.9)	
High (n = 4)	9 (1.0–17.0)	
Overall survival		
Low (n = 343)	60 (50.6–69.4)	0.077
Moderate (n = 76)	42 (24.5–59.5)	
High (n = 4)	*	
**Hepatocellular carcinoma (n = 83)**		
Time to recurrence		
Low (n = 64)	34 (0.0–74.1)	<0.001
Moderate (n = 16)	35 (7.9–62.1)	
High (n = 3)	*	
Overall survival		
Low (n = 64)	68 (46.1–89.5)	<0.001
Moderate (n = 16)	*	
High (n = 3)	2 (0.0–5.2)	
**Cholangiocarcinoma (n = 69)**		
Time to recurrence		
Low (n = 35)	29 (7.1–50.9)	0.093
Moderate (n = 28)	14 (11.1–16.9)	
High (n = 6)	8 (5.1–10.9)	
Overall survival		
Low (n = 35)	40 (35.6–44.4)	0.004
Moderate (n = 28)	16 (1.1–30.9)	
High (n = 6)	7 (0–23.8)	

* Median survival could not be estimated for the high operative difficulty group due to the small sample size and insufficient number of events.

## Data Availability

The data that support the findings of this study are available from the corresponding author, M.P., upon reasonable request.

## Supplementary Materials

The following supporting information can be downloaded at: https://www.mdpi.com/article/10.3390/cancers18030407/s1, Table S1: Operative Difficulty Calculator.
